# Determination of critical concentration for drug susceptibility testing of *Mycobacterium tuberculosis* against para-aminosalicylic acid with clinical isolates with *thyA*, *folC* and *dfrA* mutations

**DOI:** 10.1186/s12941-022-00537-z

**Published:** 2022-11-05

**Authors:** Wei Wang, Shanshan Li, Qiping Ge, Haiping Guo, Yuanyuan Shang, Weicong Ren, Yufeng Wang, Zhongtan Xue, Jie Lu, Yu Pang

**Affiliations:** 1 Department of Bacteriology and Immunology, Beijing Key Laboratory On Drug-Resistant Tuberculosis Research, Beijing Tuberculosis and Thoracic Tumor Research Institute/Beijing Chest Hospital, Capital Medical University, Beijing, 101149 People’s Republic of China; 2 Department of Tuberculosis, Beijing Tuberculosis and Thoracic Tumor Research Institute/Beijing Chest Hospital, Capital Medical University, Beijing, 101149 People’s Republic of China; 3 Innovation Alliance On Tuberculosis Diagnosis and Treatment, Beijing, 101149 People’s Republic of China; 4 Beijing Key Laboratory for Pediatric Diseases of Otolaryngology, Head and Neck Surgery, Beijing Pediatric Research Institute, National Center for Children’s Health, Beijing Children’s Hospital, Capital Medical University, Beijing, 100045 People’s Republic of China

**Keywords:** *Mycobacterium tuberculosis*, Para-Aminosalicylic Acid, Critical Concentration, Drug Susceptibility Testing

## Abstract

**Background & Objectives:**

Accurate determination of antimicrobial resistance profiles is of great importance to formulate optimal regimens against multidrug-resistant tuberculosis (MDR-TB). Although para-aminosalicylic acid (PAS) has been widely used clinically, the reliable testing methods for PAS susceptibility were not established. Herein, we aimed to establish critical test concentration for PAS on the Mycobacterial Growth Indicator Tube (MGIT) 960 in our laboratory settings.

**Methods:**

A total of 102 clinical isolates were included in this study, including 82 wild-type and 20 resistotype isolates. Minimum inhibitory concentration (MIC) was determined by MGIT 960. Whole-genome sequencing was used to identify the mutation patterns potentially conferring PAS resistance. Sequence alignment and structure modelling were carried out to analyze potential drug-resistant mechanism of *folC* mutant*.*

**Results:**

Overall, the Minimum inhibitory concentration (MIC) distribution demonstrated excellent separation between wild-type and resistotype isolates. The wild-type population were all at least 1 dilution below 4 μg/ml, and the resistotype population were no lower than 4 μg/ml, indicating that 4 μg/ml was appropriate critical concentration to separate these two populations. Of 20 mutant isolates, 12 (60.0%) harbored *thyA* mutations, 2 (10%) had a mutation on upstream of *dfrA*, and the remaining isolates had *folC* mutations. Overall, *thyA* and *folC* mutations were scattered throughout the whole gene without any one mutation predominating. All mutations within *thyA* resulted in high-level resistance to PAS (MIC > 32 μg/ml); whereas the MICs of isolates with *folC* mutations exhibited great diversity, ranged from 4 to > 32 μg/ml, and sequence and structure analysis partially provided the possible reasons for this diversity.

**Conclusions:**

We propose 4 μg/ml as tentative critical concentration for MGIT 960. The major mechanism of PAS resistance is mutations within *thyA* and *folC* in MTB isolations. The whole-gene deletion of *thyA* locus confers high-level resistance to PAS. The diversity of many distinct mutations scattered throughout the full-length *folC* gene challenges the PCR-based mutation analysis for PAS susceptibility.

## Introduction

The emergence of multidrug-resistant tuberculosis (MDR-TB) threatens the progress of global control efforts [1]. In 2020, 9.9 million persons were estimated to develop active tuberculosis (TB), of whom 465,000 were afflicted with MDR-TB [2]. Because they acquire resistance to the two most effective bactericidal agents, rifampin (RIF) and isoniazid (INH), the treatment of MDR-TB requires the use of second-line medications which are more expensive and toxic, but less effective than treatment for drug-susceptible TB [1, 3]. Despite undergoing second-line treatment for 9–24 months, this disease is associated with worse outcomes than drug-susceptible TB, and only 54% of patients achieve treatment success [2]. Accurate determination of antimicrobial resistance profiles is of great importance to formulate optimal regimens against MDR-TB [4].

Para-aminosalicylic acid (PAS) is one of the first anti-TB agents found to be effective in the 1940s [5]. It can competitively inhibit para-aminobenzoate from entering the folate pathway at the enzyme dihydropteroate synthase, thereby disrupting the biosynthesis of DNA precursor [6]. Previous findings have demonstrated that multiple genes conferring the folate biosynthesis are involved in PAS resistance in MTB, including prevention of sufficient bioactivation within the folate synthesis pathway and mitigation of target inhibition [7–9]. Of these mechanisms, the mutations within *thyA* (folate-dependent thymidylate synthase) and *folC* (dihydrofolate synthase) are the most frequently reported conferring PAS resistance in clinical MTB isolates [10, 11]. Over-expression of *dfrA* (dihydrofolate reductase) gene also conferred to PAS resistance [9, 12].

In the WHO guidelines, PAS should be used as a potential Group C drug for patients with MDR-TB [13]. Although it has been widely used clinically, the reliable testing methods for PAS susceptibility were not established. Therefore, there is an urgent need to determine the critical concentration of PAS for discrimination between susceptible and resistant strains, which will aid in better patient management of MDR-TB and in the prevention of further community transmission. To address this concern, we aimed to establish critical test concentration for PAS on the Mycobacterial Growth Indicator Tube (MGIT) 960 in our laboratory settings.

## Methods

### Bacterial strains and DNA extraction

We collected a set of 102 MTB isolates from the BioBank of Beijing Chest Hospital and Beijing Institute of Tuberculosis Control. The drug susceptibilities of MDR-TB isolates were determined by conventional phenotypical method as endorsed by WHO [14]. MDR-TB was defined as in vitro resistance to both rifampicin and isoniazid; and extensively drug-resistant TB (XDR-TB) was defined as MDR-TB plus additional resistance to both fluoroquinolone and at least one additional Group A drug. All MDR-TB isolates in this panel were subcultured on Löwenstein-Jensen (L-J) medium for DNA extraction purpose. After 4 weeks of incubation, the fresh bacteria colonies were harvested from the surface of L-J medium. The cetyltrimethylammonium bromide (CTAB) method was used to extract genomic DNA of MTB as previously reported [15]. The high-quality DNA samples underwent whole genome sequencing (WGS) using Illumina HiSeq 2000 platform. The raw sequence data were aligned to the MTB H37Rv reference genome (NC000962.3), and the single nucleotide polymorphism (SNP) and insertion-deletion (InDel) of the target genome were identified as previously described [16]. The TB-Profiler online informatics platform (https://github.com/jodyphelan/TBProfiler) was used to identified drug resistance specific mutations. The raw sequencing data was deposited on the China National GeneBank DataBase (CNGBdb) with the reference number **CNP0002824**. After a careful check of WGS data, we picked up MTB isolates with *thyA*, *folC* and *dfrA* mutations conferring PAS resistance. Additionally, a panel of non-MDR-TB isolates from TB patients who had never been exposed to PAS were included to explore the wild-type MIC distribution.

### Minimum inhibitory concentration

Pure PAS powder was synthesized by HanXiang Biotech Co., Ltd. (Shanghai, China), and dissolved in sterile water. MIC values were determined with the BACTEC MGIT 960 system [17]. For each 7-ml MGIT tube, 0.8 ml of MGIT 960 Growth Supplement and 0.1 ml of serial dilutions of the drug stock solution were added, respectively. For each isolate grown on L-J medium, a suspension of the microorganism was prepared in sterile saline at a density of 0.5 McFarland and was then diluted 1:5 with sterile saline. 0.5 ml of this inoculum was used for MGIT 960 tube containing drugs; for the drug-free growth control tube, the inoculum was diluted 1:100 with sterile saline, and then 0.5 ml was inoculated into the control tube. The drug concentrations tested for PAS included 0.25, 0.5, 1, 2, 4, 8, 16, and 32 μg/ml. The tubes were incubated at 37 °C in the BACTEC MGIT 960 instrument and automatically monitored for fluorescence development. The MIC was defined as the lowest concentration that the lowest drug concentration that maintained a growth index (GI) of < 100 at the time when the growth of the control reached a GI of > 400. Additionally, the fully drug-susceptible *M. tuberculosis* H37Rv (ATCC 27294) strain was included in each testing round. The epidemiological cut-off value (ECOFF) was defined as the concentrations of which could distinguish microorganisms without (wild-type) and with phenotypically detectable acquired resistance mechanisms (non-wild-type) to PAS according to the guidelines by the European Committee on Antimicrobial Susceptibility Testing [18].

### Sequence alignment and structure analysis

According to previous studies [6], and the activation of PAS relies on FolC to form the hydroxyl dihydrofolate. To explore the mechanism involved in the diversity in in vitro drug susceptibilities against PAS, we firstly performed sequence alignment of MTB FolC (UniProt entry I6Y0R5) with its orthologs from *Escherichia coli* (UniProt entry P08192), *Haemophilus influenzae* (UniProt entry P43775), and*Bacillus subtilis* (UniProt entry Q05865). Multiple sequence alignment was performed using ClustalO [19] and formatted in ESPript [20].

To further explore the drug-resistant mechanism caused by the mutants, the structures of wild-type MTB FolC and mutants were modelled by SWISS-MODEL (http://swissmodel.expasy.org/). Because FolC catalyzes the formation of dihydrofolate (DHF) from substrate dihydropteroate (DHP), we tried to obtain the structure of MTB FolC complexed with DHP or its analog. The MTB FolC structure (PDB file 2VOS) was superimposed to *E. coli* FolC complexed with dihydropteroate-phosphate (DHP-P) and adenosine diphosphate (ADP) (PDB file 1W78), and then the PDB file containing structures of MTB FolC, DHP-P and ADP was used as modelling template by SWISS-MODEL to obtain wild-type MTB FolC in complex with DHP-P and ADP.

The mutations could influence the protein stability and protein–ligand binding. Free energy changes were calculated by Eris [21] and PremPS [22] to predict the impact of point mutations on protein stability (*ΔΔG* < 0, stabilizing mutations; *ΔΔG* > 0 destabilizing mutations,). Structural analysis was performed only for point mutations because it is not possible to correctly calculate the free energy for stop codons and frameshifts. Due to different parameters in different methods, *ΔΔG* values of some mutations from Eris and PremPS were conflicting. Thus, we only consider the mutations with *ΔΔG* > *0* from both methods as the mutations that might influence the protein stability. Discovery Studio Visualizer v.4.5 software (BIOVIA, Dassault Systèmes, San Diego, CA, USA) was used to visualize the three-dimensional structures of proteins and the “Structure Monitor” and “Receptor-Ligand Interactions” modules were used to investigate the detailed intramolecular (between different residues within one protein molecule) and intermolecular (between protein and ligand or between different protein molecules) interactions.

### Data analysis

The sensitivity was calculated as a proportion of resistotype isolates with resistant results in total of resistotype isolates; whereas the specificity was calculated as a proportion of wild-type isolates with susceptible results in total of wild-type isolates.

## Results

### Drug susceptibility profiles

A total of 102 clinical isolates were included in our analysis. Table 1 summarizes the detailed profiles of MTB isolates with resistance to 9 drugs: INH, RIF, Streptomycin (SM), Ethambutol (EMB), Levofloxacin (LVX), Capreomycin (CPM), Kanamycin (KM), Ofloxacin (OFX) and Amikacin (AMK). Among these isolates, 82 were pas-susceptible, 56 were mono-resistant, 36 were poly-resistant, and 20 were pre-XDR-TB (defined as MDR-TB plus resistance to LVX).

**Table 1:** Drug susceptibility profiles of PAS-resistant MDR-TB and PAS-susceptible isolates

Drug resistance profile (n = 102)	Number of strains	Proportion (%)
PAS-resistant isolates (n = 20)		
INH + RIF + SM + EMB + LVX + AMK + CPM + KM + OFX	9	8.8
INH + RIF + SM + EMB + LVX + AMK + CPM + OFX	1	1.0
INH + RIF + SM + EMB + LVX + CPM + KM + OFX	1	1.0
INH + RIF + SM + EMB + LVX + CPM + OFX	3	2.9
INH + RIF + SM + EMB + LVX + AMK + KM + OFX	4	3.9
INH + RIF + SM + EMB + LVX + AMK + OFX	2	2.0
PAS-susceptible isolates (n = 82)		
INH + SM + CPM	1	1.0
SM + AMK + CPM	1	1.0
INH + SM	5	5.0
SM + CPM	4	4.0
SM + EMB	5	5.0
INH	2	2.0
SM	36	35.3
EMB	1	1.0
LVX	16	15.7
CPM	1	1.0
Susceptible to all drugs	10	9.8

*INH* isoniazid, *RIF* rifampin, *SM* streptomycin, *EMB* ethambutol, *LVX* levofloxacin, *AMK* amikacin, *CPM* capreomycin, *KM* kanamycin, *OFX* ofloxacin

### Distribution of MICs to PAS

**Fig. 1:**
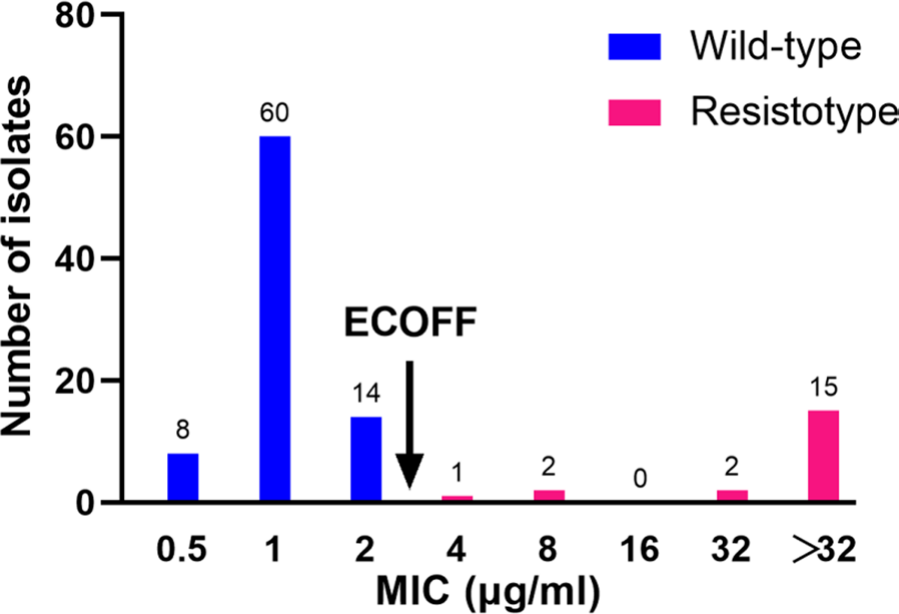
Distribution of PAS MICs (μg/ml) in the MGIT for wild-type and resistotype MTB isolates. *ECOFF* epidemiological cutoff

The overall MIC distribution determined by MGIT with doubling concentrations of PAS is shown in Fig. 1. The reference H37Rv strain had a MIC of 0.25 μg/ml. The MICs of clinical isolates ranged from 0.5 to 32 μg/ml, with a median of 4 μg/ml. For wild-type isolates, the MICs ranged from 0.5 to 2 μg/ml; whereas the 20 isolates carrying mutations within *thyA*, *dfrA* and *folC* exhibited MICs no lower than 4 μg/ml. Overall, the MIC distribution demonstrated excellent separation between wild-type and resistotype isolates. The wild-type population were all at least one dilution below 4 μg/ml, and the resistotype population were no lower than 4 μg/ml, indicating that 4 μg/ml was appropriate critical concentration (CC) to separate these two populations. The CC of 4 μg/ml yielded a concordance rate of 100% between genotypic and phenotypic PAS susceptibility, indicating that this CC identified 100% of wild-type clinical isolates as PAS-susceptible, 100% of *thyA* mutants as PAS-resistant, and 100% of *folC* mutants as PAS-resistant, yielding a sensitivity of 100% and a specificity of 100%.

### Correlations between PAS MICs and mutations within thyA, dfrA and folC

Among the 20 PAS-resistant isolates, harbored 14 different mutation types within three genes *thyA, dfrA* and *folC*. As shown as Table 2, 12 (60.0%) isolates harbored *thyA* mutations, 2 (10%) had a mutation on upstream of *dfrA*, and the remaining isolates had *folC* mutations, and the remaining isolates had *folC* mutations. Overall, *thyA* and *folC* mutations were scattered throughout the whole gene without any one mutation predominating. 4 different mutation types were noted in the PAS-resistant isolates, including 14 (70.0%) missense mutations, 2 (10.0%) small insertion/deletions and 4 (20.0%) whole-gene deletions. The most frequent mutation type was *thyA gene* deletion, followed by at + 225 (C/A). Of note, 8 novel mutations conferring PAS resistance were firstly reported in this study. Additionally, all mutations within *thyA* resulted in high-level resistance to PAS (MIC > 32 μg/ml); whereas the MICs of isolates with *folC* mutations exhibited great diversity, ranged from 4 to > 32 μg/ml.

**Table 2:** The molecular characteristics of 20 isolates carried mutations conferring PAS resistance

Locus	ID of isolates	Nucleotide changes	Amino acid changes	MIC (μg/ml)
*thyA*	22,757	G → T at 60	D20Y^a^	> 32
	25,001	del_A at 115	Frameshift^a^	> 32
	22,773	C → A at 225	H75N	> 32
	15,219	C → A at 225	H75N	> 32
	15,821	C → A at 225	H75N	> 32
	31,792	A → G at 259	T87A^a^	> 32
	17170	A → G at 411	E137G^a^	> 32
	30329	ins_C at 790	Frameshift^a^	> 32
	28422	*thyA* gene deletion	–	> 32
	28198	*thyA* gene deletion	–	> 32
	25426	*thyA* gene deletion	–	> 32
	18402	*thyA* gene deletion	–	> 32
*dfrA*	29861	C → A at -70	Upstream control element^a^	> 32
	26406	C → A at -70	Upstream control element^a^	> 32
*folC*	15821	G → C at 146	R49P	> 32
	15765	A → G at 292	S98G	32
	18069	A → C at 431	K144T^a^	8
	15010	A → G at 448	S150G	> 32
	25174	A → C at 458	E153A	4
	16003	A → C at 458	E153A	8
	14803	C → T at 1021	R341C^a^	32

^a^Mutation not previously reported

### Sequence and structure analysis of wild-type and mutant FolCs

**Fig. 2:**
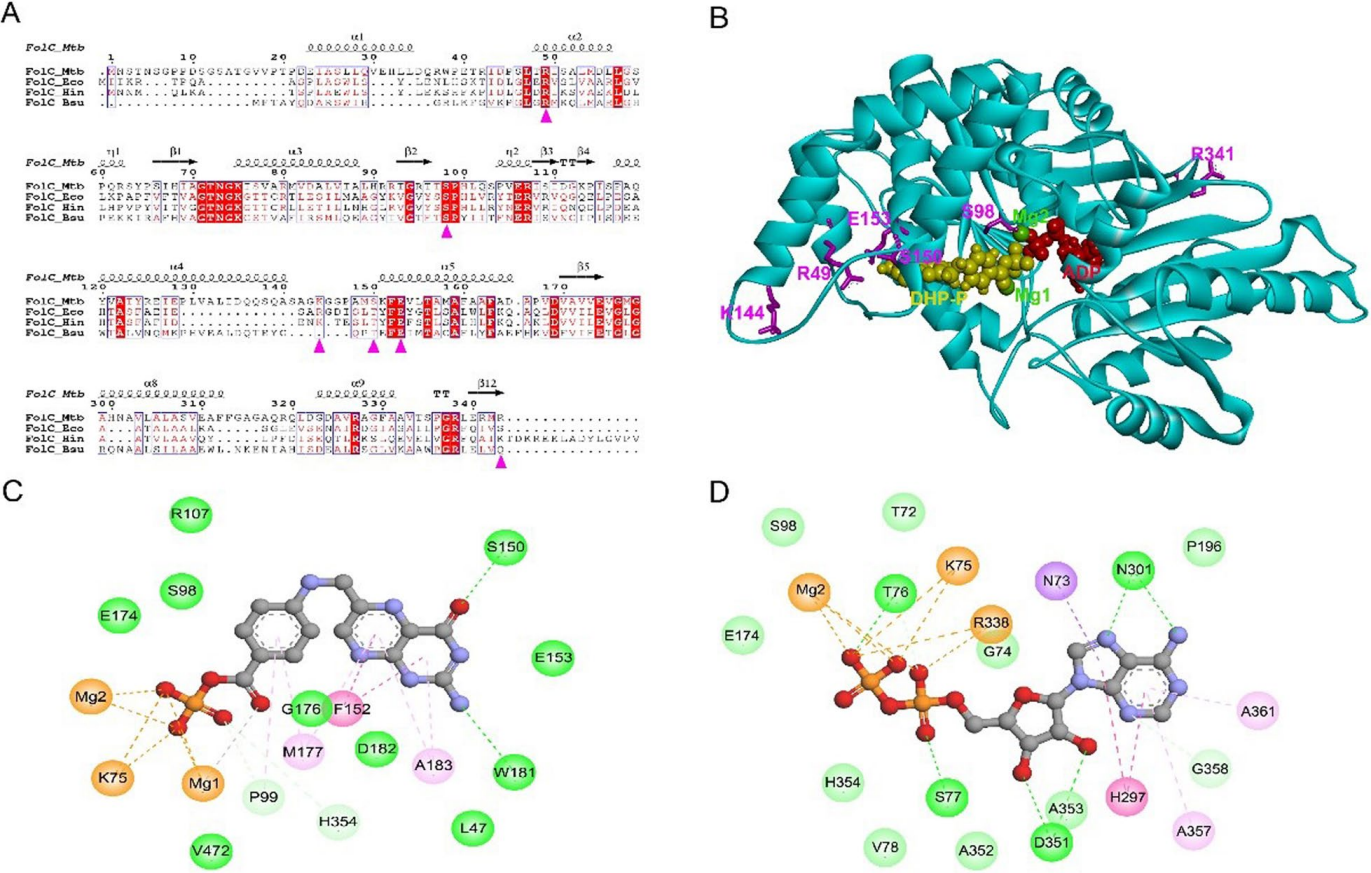
Sequence and structure analysis of wild-type FolC and mutants. **A** Sequence alignment of FolC orthologs in mycobacterial species. Magenta triangles indicated the position of mutants found in our study. FolC_MTB, FolC from MTB (UniProt entry I6Y0R5); FolC_Eco, FolC from *E. coli* (UniProt entry P08192); FolC_Hin, FolC from *H. influenzae* (UniProt entry P43775); FolC_Bsu, FolC from *B. subtilis* (UniProt entry Q05865). **B** Three-dimensional structure of wild-type MTB FolC in complex with DHP-P and ADP. The LeuRS protein (cyan) was displayed in cartoon mode. The six different residues (magenta) were shown as sticks. Ligands DHP-P (yellow), ADP (red) and magnesium ions (green) were represented in CPK mode. **C**, **D** The 2D diagram showing the interactions between wild-type FolC and DHP-P (**C**) and ADP (**D**). The ligand molecules, namely DHP-P and ADP, were shown in the middle with a display style of ball and stick. The colored balls indicated the residues involved in the direct interactions between FolC and ligand. The green, purple and yellow dash line connecting ligand and corresponding residue indicated intermolecular hydrogen bond, hydrophobic interaction and attractive charge, respectively. Residues involved in hydrogen bond, van der Waals interactions or polar interactions were represented by green balls. Residues involved hydrophobic interactions and attractive charge were displayed by purple and orange balls, respectively

In MTB, FolC catalyzes the formation of DHF from substrate DHP. After addition of PAS, FolC can catalyze the formation of the DHF analog hydroxyl dihydrofolate, which is important for the activation of PAS. Sequence alignment revealed that residues R49, S98 and E153 (in order of MTB FolC) were highly conserved in bacteria FolC, suggesting important roles of these residues for structure or function of FolC. Amino acid threonine appeared in other bacteria FolC at the corresponding position at residue S150 of MTB FolC, indicating the hydroxyl group on the side chain was important for this position (Fig. 2A). The results from both Eris and PremPS indicated four mutants of FolC with high-level resistance to PAS, namely R49P, S98G, S150G and R341C, would lead to structure instability (Table 3).

**Table 3:** The effect of mutations on FolC protein stability

Amino acid change	MIC	ΔΔG (kcal/mol) ^a^	
		Eris	PremPS
R49P	> 32	2.91	1.27
S98G	32	1.39	1.13
K144T	8	3.89	−0.01
S150G	> 32	1.67	0.73
E153A	8	−1.13	0.44
R341C	32	1.79	0.38

^a^The free energy (ΔΔG) was calculated for point mutations in the available protein structures by using two endpoint methods, namely, Eris and PremPS. ΔΔG < 0, stabilizing mutations; ΔΔG > 0, destabilizing mutations

Because FolC catalyzes the formation of DHF from substrate DHP, we built the structure model of MTB FolC complexed with DHP analog DHP-P and ADP. As shown in Fig. 2B, residues R49, S98, S150 and E153 located very close to DHP binding site, while residues K144 and R341 were far away from the catalytic site. Detailed analysis of protein–ligand interaction showed that S98 and E153 had van der Waals interaction with DHP-P, and the hydroxyl group on the side chain of S150 formed a hydrogen bond with DHP-P (Fig. 2C). Only S98 had van der Waals interaction with ADP.

Residue R49 located on α2 helix of FolC, and R49P mutation probably disrupted this α-helix, leading to destruction of secondary structure of FolC. In addition, R49P mutation lost the positive charge of Residue 49, leading to loss of electrostatic attractive interaction between R49 and E153. Residue S98 was close to the interface of DHP-P and ADP and had a hydrogen bond with magnesium ion, which was important for the catalyzation process. S98G mutation lost the hydroxyl group on the side chain, which might change the local catalytic environment. S150 formed a hydrogen bond with DHP-P, and S150G mutation led to loss of this intermolecular hydrogen bond due to loss of side-chain hydroxyl group. K144T mutation did not disturb the interaction between FolC and DHP-P or any intramolecular interaction within FolC. E153 had electrostatic attractive interaction with R49, and E153A mutation disrupted this electrostatic interaction. R341C mutation did not disturb any intramolecular interaction within FolC or intermolecular interaction between FolC and any ligand.

## Discussion

The individualized DST-guided therapy plays a particularly crucial role on clinical outcomes of MDR-TB patients [1]. The major challenge in conducting phenotypical DST is the lack of critical concentrations of second-line drugs for resistance testing [14]. In this study, we firstly established a tentative critical concentration for PAS between the highest MICs of known wild-type isolates and the lowest MICs of known resistant isolates. A recent study by Dusthackeer and coresearchers revealed an ECOFF of 1 μg/ml for PAS by using the Sensititre MYCOTBI plate [23], which varied from the observation in the present study (4 μg/ml). A plausible explanation for this difference is that MICs are method dependent. Although this difference exists, our data clearly demonstrated that this value could accurately distinguish between PAS-susceptible and resistant populations. Recently, the CLSI recommend the analysis of PK data to provide a more precise definition of critical concentration [24, 25]. With the licensed dosage of 8 g, the average plasma concentrations reach 50–100 μg/ml [26], which was remarkably higher than the ECOFF concentration observed in our study. The great gap lying between ECOFF concentration and plasma concentration might be linked to suppression of resistance emergence. Despite the long history of PAS usage, previous study by Deng and colleagues revealed that the prevalence of PAS resistance was significantly lower than other second-line drugs in XDR-TB isolates [27]. Similar results were found in our XDR-TB cohort in Beijing (data not shown). These surveillance data may indicate the low occurrence of PAS resistance after exposure to this drug, which could be partly explained by its high peak concentration (C_max_) to MIC ratio that suppresses the resistance development in companion with other drugs. In view of these results, we preferred to propose the most conservative cut-off of 4 μg/ml for the MGIT culture system. Further studies are urgently required to validate our results in clinical trial data.

Mutations within *thyA* gene were previously identified in in one-third of the PAS resistance clinical isolates and laboratory mutants [7, 11]. In this study, missense mutations, small insertion/deletions and whole-gene deletions within *thyA* locus were identified in PAS-resistant isolates. Of note, all isolates with *thyA* mutations exhibited high-level resistance to PAS (MIC ≥ 64 μg/ml), indicating a major contribution of thymidylate synthase that catalyzes the activation of PAS into its active form in PAS resistance. In a recent genome-wide analysis of MDR-TB isolates, Coll and colleagues found that five isolates across four countries contained large *thyA* deletions of varying length [28]. Similarly, we also recorded four samples harboring whole-gene deletions in *thyA* loci, which were associated with high-level PAS resistance. This potentially lethal deletion is permissible in MTB due to the presence of the complementary function homologue ThyX [29].

Structure analysis of FolC provided probable explanation for different resistance levels of some mutants. Previous study revealed that a four-helix bundle (α1 to α2/α4 to α5) of FolC was important for interaction with DHP [30], and mutations R49P, K144T, S150G and E153A in our study were located in this helix bundle. The destruction of α2 helix by R49P mutation not only resulted in structure instability but also possibly influenced the binding pocket of DHP, which could be the probable reason for high-level resistance to PAS of R49P mutant. It is likely that the high-level resistance of S150G mutants was due to decreased binding affinity of DHP caused by loss of the intermolecular hydrogen bond between FolC and DHP. The probable reason for low-level resistance of E153A mutant was that this mutation only disturbed the local charge interaction. For S98G mutant, the mutation led to loss of hydroxyl group on its side chain and might influence local catalytic environment due to its special position close to the interface of DHP and ADP.

We also acknowledged several limitations to the present study. First, despite the enrollment of mutants conferring PAS resistance by WGS, the small sample size of PAS-resistant isolates may weaken the significance of our conclusion. Second, previous studies revealed the presence of intra- and interlaboratory variations of PAS MIC determinations [31]. Unfortunately, all experiments were only performed in a single laboratory, which highlights the need to validate our proposed critical concentration for PAS in a panel of strains across different laboratories. Third, besides *thyA* and *folC*, multiple genes have been reported to be associated with PAS resistance, including *ribD*, *folP1* and *folP2* [32, 33]. However, no mutations were identified in these loci among our MDR-TB isolate. This may be related to the low frequency of *ribD* and *dfrA* for PAS resistance. Finally, previous studies have demonstrated the MTB lineage-specific drug resistance evolution [34, 35]. However, the predominance of Beijing genotype strains hampered us to investigate the potential association of MTB lineage with drug resistance in our cohort.

In conclusion, we propose tentative critical concentration for MGIT 960 between the highest MICs of known wild-type isolates and the lowest MICs of known resistant isolates. The major mechanism of PAS resistance is mutations within *thyA* and *folC* in MTB. The whole-gene deletion of *thyA* locus confers high-level resistance to PAS. The diversity of many distinct mutations scattered throughout the full-length *folC* gene challenges the PCR-based mutation analysis for PAS susceptibility. Further studies are urgently required to validate whether the proposed critical concentration could predict clinical outcomes in cohorts of patients with MDR-TB.

## Data Availability

The raw sequencing data was deposited on the China National GeneBank DataBase (CNGBdb) with the reference number CNP0002824.

## Abbreviations

PAS: Para-aminosalicylic acid
MIC: Minimum inhibitory concentration
MDR-TB: Multidrug-resistant tuberculosis
TB: Tuberculosis
RIF: Rifampin
INH: Isoniazid
CTAB: Cetyltrimethylammonium bromide
SNP: Single nucleotide polymorphism
MGIT: Mycobacterial Growth Indicator Tube
GI: Growth index
ECOFF: Epidemiological cut-off value
DHP: Dihydropteroate
DHP-P: Dihydropteroate-phosphate
ADP: Adenosine diphosphate
SM: Streptomycin
EMB: Ethambutol
LVX: Levofloxacin
CPM: Capreomycin
KM: Kanamycin
OFX: Ofloxacin
AMK: Amikacin
CC: Critical concentration
C_max_: High peak concentration
